# Out of Time: Adolescents and Those Who Wait For Them

**DOI:** 10.1080/0075417X.2021.1954977

**Published:** 2021-05-04

**Authors:** Jocelyn Catty

**Affiliations:** Tavistock & Portman NHS Foundation Trust, 120 Belsize Lane, London NW3 5BA

**Keywords:** Crisis, risk, temporality, adolescent mental health, deliberate self-harm, suicide, adolescence, psychoanalytic psychotherapy

## Abstract

This paper draws on the scholarship of an inter-disciplinary project about time and waiting in healthcare to explore questions of urgency and risk in clinical work with depressed and suicidal young people, and how the feeling of being compelled to act can be meaningfully explored from a psychoanalytic perspective. The paper examines adolescence as both a time of inherent crisis and one in which self-harm and suicidal ideation represent particular challenges. It then considers Child and Adolescent Mental Health Service practice in relation to acute mental health crisis, and in the context of the chronic crisis affecting the UK National Health Service. Considering both formal psychoanalytic psychotherapy and the contribution of psychoanalytic thinking to multidisciplinary discussions and emergency work in CAMHS, the author then considers the anticipatory anxieties that affect such work, and the particular role of psychoanalytic thinking for young people burdened by suicidal ideation and the professionals caring for them.

And I will show you something different from either Your shadow at morning striding behind you Or your shadow at evening rising to meet you; I will show you fear in a handful of dust.

T. S. Eliot, The Waste Land (1922), ll. 27-30

In this paper, I shall be exploring the implications of ideas about time and waiting in relation to psychoanalytic psychotherapy with young people, examining the significance of adolescent crisis and the twin shadows of suicide and deliberate self-harm. The paper arises out of my association with an inter-disciplinary medical humanities project, *Waiting Times*, a five-year endeavour exploring the concepts of *delay* and *impeded time* in relation to practices of care that may be regarded as *waiting with* or *waiting alongside* [1]. By drawing on the scholarship of this larger project,I aim to use thoughts about temporality, care and crisis to illuminate areas that I have found to be central to clinical work with young people in crisis. I use psychoanalytic thinking about adolescence to consider the temporal nature of mental health crisis for young people. I then examine the various anticipatory anxieties attendant upon working with young people in crisis, exploring these in the wider context of the *chronic crisis* of the UK National Health Service (NHS; Baraitser & Brook, 2020) and the specific context of the time- and crisis-management strategies of contemporary Child and Adolescent Mental Health Services (CAMHS).

Conflicts over time have come to seem characteristic of the modern world. Accounts of time and pace cite, *inter alia*, the development of faster forms of transport in the twentieth century, and of digital technology in the twenty-first, as evidence that our lives are accelerating. This produces an urgency about the idea of somehow slowing things down, while the sense of a world careering towards an unpredictable, if not catastrophic, future also prevails. Even before the obvious temporal crisis of 2020-21 - the apparent suspension of time through the use of lockdown in response to the Covid-19 pandemic to which our project has responded in a collection of papers [2]-the sense of a crisis in and of time was evident. Lisa Baraitser has argued that these phenomena give rise to experiences in which waiting and delay predominate:

Despite an ever-increasing sense of acceleration, because the spooling of time towards a possible future seems to have come unravelled in the contemporary moment, experiences of interruption, suspension, delay, and slowness strongly insist in affective life(Salisbury & Baraitser, 2020, p. 104; see also Baraitser, 2017).

Born in the years following the turn of the 21^st^ century, a moment surrounded by predictions of the ‘end of days’ (whether the end of digital time as the world’s electronic clocks imploded and systems ground to a halt, or with more biblical implications), today’s young people approach adulthood amidst a flurry of drastic predictions: unlikely, apparently, to be able to find work, buy a home, or even, perhaps, retire. While these concerns continue to be mediated by class, race and gender, they now also intersect with age, to give an impression of a generation pre-emptively disadvantaged. Young people thus find themselves at the maelstrom of a culture in which the relentless pace of modernity and technology keeps them forever active, yet waiting for an uncertain future. It is perhaps not surprising, then, that increasing rates of deliberate self-harm or non-suicidal self-injury are reported (Therrien, 2018), as well as hospital admissions for self-harm (Nuffield Trust, 2019). Blame is often laid upon an increasing emphasis on academic performance (Waddell, 2018, p. 40), and on social media (Karim, Oyewande, Abdalla, Ehsanullah, & Khan, 2020; Kelly, Zilanawala, Booker, & Sacker, 2018; Royal College of Psychiatrists, 2020) and its insistence on the impossibility of waiting (Wajcman, 2015). Young people with depression and other forms of emotional disturbance seem either to slow time down into a kind of suspended animation, or impel it forward with ever-more-frantic distortions of the ordinary *sturm und drang* of adolescence, such as suicidal acts. How are we to understand these contradictory responses?

I shall seek here to examine the unique temporal pressures on young people, which render adolescence always, in a sense, a crisis of time; and to revisit questions of how the current mental health crisis can be understood in this context. I shall argue that adolescent crisis may represent a protestation against the seemingly relentless temporal thrust towards the future: an attempt to halt progress, even at a terrible cost. I shall think about the ways in which psychoanalytic psychotherapy within the NHS – an offer of time which is always already time-bound by the demands of a public health system – can operate within the time structures associated with crisis, and the nature of its particular contribution. This is not a paper about completed suicide and its tragic impact on family, friends or professionals. Instead, I shall try to consider the pressures of anticipatory anxiety – the shadow of suicide – on psychoanalytic work, in the context of urgency and risk. First, however, I consider the nature of the mental health crisis in relation to time, and the particular significance of adolescence as a critically transitional state.

## Navigating time: adolescence and crisis

When a mental health crisis arrives, it may force a collapse of both time and space. I once attempted to interview a woman in her early thirties in a day centre for people with chronic mental illness:

‘Mary’ spoke with a fluidity that left me grasping at words (which I later learned to call ‘florid’). She seemed to be at once with me and miles away. When her attention seemed more focused, I asked how she had come to be using this day centre. She paused. ‘Well’, she hesitated, ‘really, it was navigation errors.’ She began to speak again, faster and faster. After a while, a phrase leaped out at me: ‘Cathy come home’.

*Cathy Come Home* (Sandford & Loach, 1966), the 1966 television play about homelessness directed by Ken Loach, had topped the polls for best television drama during the period when I met Mary, around the year 2000, and is widely regarded as having made a crisis visible (indeed, the homelessness charity Crisis was launched in response to it). Mary’s communication seemed to me then, as it does now, to conjure up a world of meaning about the ‘navigation errors’ that had dislocated her in, perhaps, both space and time. Her physical location in a London day centre in 2000 seemed to represent her having come unstuck. Perhaps she too felt very far from home: either the physical home of her birth, her temporal origins in 1966 (when perhaps she might have been born), or the different location in which she might fantasise that her life might otherwise have unfolded; far away, too, from her mental equilibrium, her internal world set badly adrift.

We might read this against Michael Flexer’s (2020) description of psychosis as an ‘explosion through which the self can be lost in time’ (p. 457). Flexer’s inter-disciplinary account of the mental health crisis utilises the concept of the Event, coined by philosopher Gilles Deleuze (2004):

The Event…is both timeless…and also a moment of pause or caesura… It serves to both fracture and construct the self, and the temporality constitutive of and by that self. (Flexer, 2020, p. 457)

Flexer argues that the event of psychosis removes the subject from a sense of narrative time: ‘the trauma *of* psychosis (which may or may not be the aetiological trauma *prior* to psychosis) violates the fixity of…relationships constitutive of communication’ (p. 458). Psychosis, in this reading, is a disruption of narrative continuity. Yet the importance of narrative continuity – its disruption precipitating crisis – may not only be relevant to psychosis. Indeed, a variety of theories postulate narrative as central to identity and mental health. Attachment theory, for instance, takes as its gold standard measure the Adult Attachment Interview (AAI), which assesses the interviewee’s attachment stability through their capacity to narrate as particularly focused on stories of loss; consistent with Flexer’s thesis, the AAI is known to be less effective for populations with psychosis (Turton, McGauley, Marin-Avellan, & Hughes, 2001). The mental health crisis, then, both produces and is produced by a crisis in and of time.

Yet what if adolescence itself could be seen as a crisis of time? While it is not alone as a key transition point in which we are pulled backwards as well as forwards (new parenthood would be another: Baraitser, 2009), perhaps in no other period of life is development so fluid, while the pressure to conform to a particular set of temporal circumstances – the task of growing from child to adult - is so strong. Yet this contrasts with the social construction of the threshold of adulthood (in prevailing Western culture, considered to be the 18^th^ birthday) as a ‘caesura’ – analogous to both the Freudian caesura of birth (Freud, 1926, p. 138) and perhaps the Deleuzian Event or crisis mentioned above - underpinned by the way both mental health and social services separate services for ‘children’ from those for ‘adults’. This construction of the 18^th^ birthday as a uniquely significant event might indeed ‘both fracture and construct the self’, as Flexer describes. The recent emphasis in public health on the transition from children’s to adults’ services, attempting to mitigate the effects of such a caesura, attests to the power of the 18^th^ birthday as a crisis point, both in service provision and symbolically.[3] While CAMHS has largely adopted the term ‘young people’ over ‘adolescents’ (in line with sociological childhood studies; see Brannen & Nilsen, 2002), the psychoanalytic emphasis on the particularity of adolescence remains valuable here, not least because it opens up an understanding of the reworking of infantile drives, traumas or dynamics in this transitional state, and enables us to consider how the pull of the future is coloured by unconscious phantasy and the pull of the past.

There are opposing narratives around young people’s mental health. One often hears the claim, ‘he’ll grow out of it’: sometimes from the lips of an anxious parent, sometimes utilised by the professional. Professionals are, after all, wary of pathologising a young person unnecessarily; what degree of disturbance is seen as developmentally appropriate and what degree pathological is a central issue. We are told, at the same time, that ‘early intervention is key’: indeed, this claim is substantiated by a wealth of research. One study found that untreated depressive difficulties in 14-year-olds are the biggest predictor of having clinical depression by the time young people reach 17 years of age (Neufeld, Dunn, Jones, Croudace, & Goodyer, 2017); another, the Tavistock Adult Depression Study (Fonagy et al., 2015), found that for the vast majority of the severely and chronically depressed adults who participated, their difficulties had started during their teen years.

Which narrative is more relevant to an individual young person is not typically easy to determine. Margot Waddell encapsulates a psychoanalytic understanding of this period of life:

The diagnostic picture is so very difficult to establish at this transitional time when the ways of defending against characteristically intense confusion are so varied and often so very extreme. Development runs unevenly, and everyone goes through adolescence in their own way and at their own speed. (Waddell, 2018, p. 177)

This conception of adolescence is one that builds on the ideas of transition, development and flux. Yet keeping to one’s own speed is almost impossible for many young people, who, Waddell writes, are ‘especially aware of how clock-time ticks on in the context of social and educational constraints, ones driven by examination requirements and the general target-setting of external culture that threatens, but also challenges, the youngsters’ natural habitats’ (p. 236). The clock is also a bodily clock, in both biological and culturally constructed ways: puberty arrives increasingly early, and the teenaged body propels changes, pushing the young person into experiences for which they feel psychically unready. Attacks on a body that feels hated or alien may ensue.

The following composite vignette [4] fashions a young woman not unfamiliar to those working with adolescents in crisis:

Jane, 16, described herself as ‘numb, most of the time’. She could not explain why she had taken an overdose of 30 Paracetamol tablets washed down with vodka, although she could intermittently access some anger at it not having ‘worked’. While her therapist could imagine, based on what was known of the family history and circumstances, that Jane might feel bleak about impending adulthood, Jane herself could not imagine being any older than she already was. Jane’s external life seemed to have ground to a halt: she had withdrawn from school, extra-curricular activities and friends, and refused to participate in family outings. Yet the fervent energy she put into carving cuts into her arms and thighs told a different story from the quiet demeanour that was more available for others to observe.

The spectacle of a teenager dropping out of school or valued activities, seemingly rejecting life, evokes the idea that time has been suspended or the sufferer paralysed.

This idea has a long history. Laura Salisbury and Lisa Baraitser (2020) suggest that the understanding of melancholia and, later, depression, in twentieth century Europe ‘came to be focalized through particular ideas and sensations of stuck, suspended, impeded, or ungraspable time that shaped and continue to mould the contours of the temporal landscapes and psychological imaginaries of (late) modernity’ (p. 105). They highlight how the insights of phenomenological psychiatrist Eugène Minkowski, that in depression ‘immanent time’ (that is, internal, inherent time) ‘seems to slow down remarkably, even to stop’ (1970, p. 332) can be traced as far back as the seventeenth century (e.g. Burton, 1621). They also cite psychiatrist Erwin Straus, who in 1928 described depression as a failure of time to flow:

When depression brings internal time to a standstill, there is no longer the possibility of resolving experiences… by stepping on into the future. Inner experience has reached an impasse. (Straus, 1928/2012, p. 211)

They draw attention to the conflict between time that is affectively experienced as paralysed or suspended, and time that is felt to proceed relentlessly:

Whether it is a sluggish over-abundance of time that does not pass, then, or an excess of time that cannot be contained but speeds by like a freight train mowing down the person standing helplessly in its path, depression here tracks modernity’s anxious relation to a temporality repeatedly imagined as having fallen out of phase with the proper rhythms of human life. (Salisbury & Baraitser, 2020, p. 112)

One of Minkowski’s patients is quoted as capturing this conflict:

I feel displaced in relation to life. I feel time flee, but I don’t have the sensation of following the movement; I have the feeling of turning in the opposite direction than the earth. (Minkoswski, 1970, p. 332)

While these accounts consider depression both as a clinical phenomenon and in history, there is an opportunity here to examine the particular implications of such ideas for young people, given their unique developmental and temporal position. Adolescence, as understood by psychoanalysis, locates young people as critically poised between the past of childhood and the future of adult life. As Waddell (2018) writes:

I think of this group as being, essentially, Janus-faced - that is, looking both from the present to the unknown and therefore unsafe future, and also from the present back to the past. (p. 199)

While adolescence may thus already be considered a temporal crisis, I would argue that for young people dealing with family or peer-group disturbance, parental mental ill health, personal or intergenerational histories of loss or trauma - all of which are implicated in the aetiology of adolescent depression (Cregeen et al., 2017) - or indeed the sense of a future already cancelled or foreclosed, the result may be even more critical. In the context of past and present trauma, the shadow of the future - catastrophic or simply unimaginable - drives some into a suspended state of withdrawal, others into frenetic activity, including drug and alcohol use, self-harm, suicidal acts or even completed suicide. Their ‘shadow at morning striding behind’ them is matched, terrifyingly, by their ‘shadow at evening rising to meet’ them (Eliot, 1922, ll. 28-29).

The concept of depression as a paralysis thus fails to do justice to the drama of adolescent life, to the whirlwind of peer or family conflict or self-harm - and to adolescent rage. That psychiatric diagnostic criteria for depression fail to acknowledge rage has been suggested by the findings of a large-scale qualitative study, IMPACT-ME (Midgley, Ansaldo, & Target, 2014), which interviewed 77 young people participating in the randomised controlled trial, IMPACT (Goodyer et al., 2016), exploring their experiences of depression, among other things (Midgley et al., 2015). The researchers found that the diagnostic criterion of ‘irritability’, such as used in the Diagnostic and Statistical Manual (American Psychiatric Association, 2013), scarcely does justice to the fury reported by the young patients in the study:

Hayley (17)… spoke of the way in which she would ‘punch walls and nearly break my knuckles because I was angry, but I didn't want to take it out on anyone else’… and immediately went on to say that she had been slicing her wrists and had scars as a result - as if the anger towards others and herself were somehow linked, where self-harm was an outlet for this. (Midgley et al., 2015, p. 274)

Rage here appears as a significant part of the experience of depressed or troubled young people. We might ask whether a key element of this rage may be against the apparently relentless march of time that propels the adolescent towards a troubled, uncertain, or even apparently cancelled future, perhaps even while they feel frozen in a traumatic past. This would suggest that young people who experience this march as unbearable may be attempting to stop time altogether, whether in fantasy or reality, through acts of suicide or deliberate self-harm.

It is striking how often young people in crisis use ideas about time to demarcate their sense of urgency or manage these temporal pressures. It is not uncommon for those who express suicidal ideation to say that they intend to die on or before their next birthday, or before a particular birthday awaiting them in the future. The 18^th^ birthday may in particular be imbued with such an idea. Other deadlines may seem less obviously symbolic but are usually full of meaning for the individual, such as needing to die before an important exam, or on the anniversary of a painful family loss. Conversely, I met one young man who had set a date for his death that was chosen precisely because he regarded it as lacking any significance for his family: his death, in this fantasy, would *not* be linked by date to any family birthday or other significant date, perhaps enabling this date, like the boy himself, to be gradually lost to memory. How are professionals to understand such alerts?

One Thursday afternoon, a crisis team is slowing to a halt before the end of the working day, tidying up paperwork and making tea. A call comes in from a school nurse: she is sitting with a 14-year-old boy who says he is going to kill himself on Monday. I experience a shocking double-reaction: ‘he’s going to kill himself, we need to get him in [to see us]; oh fine, we’ve got till Monday’. The contrast is breath-taking. After painstaking discussion with colleagues, the boy’s mother, the school nurse and the boy himself, he is escorted home, with a CAMHS appointment booked for the next morning.

These strategies to manage time’s pressure (if such they be) may extend to the array of fantasies around the impact of the young person’s death on others, including what Donald Campbell and Rob Hale call the ‘revenge fantasy’, where ‘the frequent conscious thought… is, “They will be sorry”’ (Campbell & Hale, 2017, p. 44). This is the fantasy of a future time which the young person will observe, and in which they will be vindicated. We might see this as an effective dislocation in time that puts the young person into a liminal relationship with their own being: withdrawn from the fast pace of external life and developmental time, effectively fast-forwarded to a future time (that of post-suicide) that is always just beyond the horizon. This is stasis, indeed, but a stasis forever teetering on the brink of destruction.

I am suggesting, then, that adolescence itself should be regarded as a temporal crisis, a crisis in and of developmental time; the pressure on the individual to fall into step with a relentless march forward, while also being in the grip of the conscious and unconscious reworking of earlier traumas, is perhaps unparalleled. Where this process is felt to be particularly painful, demanding, or simply impossible, the crisis may be enacted in an extreme way. To help explore how psychoanalytic thinking can offer help in such critical situations, I shall consider the implications of its offer of time in situations of urgency and crisis. First, however, I shall set the scene by thinking about crisis time against the backdrop of an NHS in *chronic crisis*.

## Acute and chronic crisisin the NHS

There is a general consensus that the NHS is in a state of *chronic crisis*: ‘an ever deepening, ongoing and enduring crisis due to chronic underfunding, creeping privatization, and a withdrawal from Europe that will lead to further staff shortages, demoralization and burnout’ (Baraitser & Brook, 2020). Lisa Baraitser and William Brook point, however, to a conflict between contrasting conceptions of crisis:

In one narrative it appears that ‘crisis’ is precisely what may bring about change, an upturn in the fate of the nation’s health service and the preservation of its attachment to the ideal of the welfare state. In the other, crisis cannot bring about change. It sets in as a permanent elongated ‘chronic’ condition, one that comes to define the temporality of healthcare itself. (p. 232)

Whether a crisis can be productive of change rather than stasis is also a key clinical question, and one to which I shall return.

Baraitser and Brook emphasise too, in their analysis of general practice, the ways in which the public nature of crisis links the individual and the systemic:

Crisis cannot always be contained within the consulting room - it has a tendency to ‘spill’ because it is affectively charged, full of anxiety, uncertainty, anger and despair. As distress becomes public, the relation between individual and systemic crisis is less easy to keep apart. (p. 243)

A backdrop of anxiety in the NHS, then, may permeate systems and staff, whether through the depletion of resources (and concomitant pressure on time and waiting lists) or through the public or private battles that flare up over the value of time. Work within the *Waiting Times* project highlights the ways in which different parts of the NHS system, both currently and historically, privilege some kinds of time at the expense of others. Stephanie Davies examines the temporal demands exerted by clinical governance in the NHS and the insidious ways in which it values administrative tasks above the clinical encounter. She questions ‘how the time of illness… [is] to be *thought* in general practice when illness is reproduced at scale as a roving statistic for which no time has been factored in?’ (Davies, 2019). Martin Moore explores the history of the appointments system in general practice, arguing that ‘appointments were intricately intertwined with emotional and psychological life’ (Moore, submitted). He traces how both GPs and patients regarded the new appointments system in the 1950s as valuing their own time more highly:

Appointment systems offered GPs a means to reclaim some of this (imagined) lost status relative to their increasingly-less deferential patients. Perhaps ironically, patients initially gave significant support for new arrangements … Where reasons were given, … reduced surgery waiting and the capacity to plan other tasks around the fixed appointment were prominent. (Moore, submitted)

Turning to CAMHS, one might point to the contrast between the orderly time of routine appointments and the crisis time of emergency work, as a site for visible and invisible battles over the value of time. While missed appointments are familiar to all those offering psychotherapy to young people, as the latter test their room to manoeuvre in relation to adult control, parents remind us of the value of paid time, or time needed to attend to the rest of the family, against the demands of the clinic. Thus, a common scenario in CAMHS occurs where an urgent referral is made, an appointment is offered, and the parent responds that the time is inconvenient: could sessions not be offered out of office hours or at the weekends? Clinicians typically respond with indignation at parents who have apparently failed to appreciate the urgency of the situation. Yet some of this urgency lies within the system, which may be able to bend *so* far (an urgent appointment within days or even hours), but no further (the evening appointment desired by a working parent being out of the question). What effect might this valuation of one kind of time over another have on the management of urgency and crisis in CAMHS?

CAMHS services are often structured around notions of urgency that may be conceptualised as operating on both vertical and horizontal axes. On the horizontal axis, services may be split into specialisms, some of them time-related (‘Adolescent’ indicating urgency as well as age; Generic teams being non-urgent; and Looked After Children teams more likely to embrace the need for ongoing care in chronic situations). On the vertical axis, we find triage systems that prioritise referrals using a ‘traffic-light’ system of red for urgent, amber for moderate and green for non-urgent cases, with young people referred from Accident and Emergency (A&E) departments trumping other referrals as the most ‘red’.

This hierarchy of risk is designed to be patient-centred, based on the urgency of the patient’s need. In practice, however, it conflates individual and organisational need, by managing clinical time and space as well as attending to the patient’s distress. A patient who has presented to A&E and who has, if necessary, received appropriate medical attention, is no longer in an emergency situation: he or she is just in an emergency *setting*. One might say, in fact, that this is a problem of location rather than time: he or she is safer than, say, a young man at home described as talking about suicide and potentially needing professional attention. Yet the latter is usually regarded as a lower priority.

Within this temporal hierarchy of *risk* (the likelihood of self-harm or suicide), the clinical management of the struggling patient rests on interpreting the meaning of the young person’s words and acts. How metaphorical, for instance, is the 13-year-old’s claim, ‘I want to kill myself’ or ‘I don’t want to live’? Is it more metaphorical when uttered by an 8-year-old? Then there is the idea that ‘actions speak louder than words’. This puts the young person who has attempted suicide by means that would be likely to be effective at the top of a hierarchy of risk: above someone who took, for instance, a smaller overdose; above someone professing suicidal intent but without any recent or historical attempts. Cutting across this hierarchy are different considerations, such as impulsivity and intentionality, not always well understood:

A senior psychiatrist delivered a training session to medical students about suicide. The students were presented with a vignette about a young man who threw himself in front of an approaching train. The man had survived, owing to falling between the rails. The students described his act as a ‘cry for help’. (Tom Burns, personal communication)

These medical students would have learned a lesson about assessing intentionality: surviving jumping in front of a train is extremely unusual. Yet this also exemplifies the power of such expressions and how they can numb thinking. Some suicidal or dangerous acts may consciously or unconsciously be the result of a motivation to elicit help, but the expression layers up meaning until it acquires another: ‘a cry for help and not, therefore, a cry of distress’.

In turn, professionals develop linguistic systems, such as the idea of *riskiness*, which patients pick up on. ‘I feel unsafe’ from a young person in CAMHS signals an array of meanings and questions: are they self-harming or considering it?; planning a suicide or tempted to? Professionals then utilise an array of signs and signals to construct a *safety plan*, such as another version of the traffic-light system, this time for the individual patient to use with his or her parents: where amber indicates a burgeoning crisis, and red an alert to the parent to contact crisis services. The *safety plans* and *risk assessments* produced by this activity are all discussed with the young person and their parents and documented, as evidence that the risk is safely contained. I began this paper with lines from *The Waste Land*; but the line that comes to me sometimes in negotiating these conversations and documents is the later line in the same poem: ‘these fragments I have shored against my ruins’ (l. 430).

Into these discussions creep ideas about waiting, as the other side of the coin of urgency and swift action. Despite the language of *zero tolerance* creeping into strategies such as those inspired by the Zero Suicide initiative in the US [5], ideas about waiting and whether or not it is manageable proliferate in increasingly under-resourced CAMHS services. ‘S/he can wait’ (if not ‘risky’). ‘If this young person waits, s/he will become risky’. ‘This young person hasn’t done anything [risky] so we can afford to wait’ (but - one might ask - what if he surprises us?). A potentially catastrophic outcome hides behind these themes and CAMHS teams must find ways of managing the attendant anticipatory anxieties.

## Psychoanalytic thinking and the shadow of suicide

To work with young people who are self-harming, who are struggling with suicidal preoccupations, or who have previously attempted suicide, is always, then, to work in the shadow of potential catastrophe. Although I have referred to the anxiety surrounding such work as ‘anticipatory’, this fails to do justice to the complex layering of conscious and unconscious anxiety involved. Anticipatory anxiety has been defined as ‘anticipatory cognitive, affective, and behavioral processes executed to avoid or reduce the impact of a potential threat’, which may be maladaptive where not ‘commensurate with the likelihood and severity of threat’ (Grupe &Nitschke, 2013, p. 488). Yet Wong (1999) points out that *signal anxiety*, the Freudian concept most closely related to this term, needs to be regarded not as an affect (consciously experienced) but as ‘a subset of unconscious mental processes that have a signal function of anticipating danger… that includes responses to both real and imagined (neurotic) appraisals of a situation’ (p. 817). Clinicians managing risk, I would argue, experience both the affect of anxiety, as they attempt to pre-empt a future ‘risk event’ such as a suicide attempt, and unconscious signal anxiety which may manifest itself in the array of measures relied upon to ‘manage’ such risk. Awareness of the fantasy in the risk management – the fantasy that a suicide can be prevented by writing a safety plan, for instance – may increase the anxiety, whether conscious or unconscious.

The shadow of suicide, then, contributes to a feeling that is more closely related to Wilfred Bion’s ‘nameless dread’ (1962, p. 116) in the way it sits behind our treatment plans, our safety plans, or our multidisciplinary discussions. While one might argue that this dread is not, in fact, ‘nameless’, it may be the unconscious elements of the fear – the failure to feel, let alone articulate fully, the fear of the young person’s suicide – that give the experience this quality. Nameless dread, after all, is the product of a failure of containment and maternal reverie which ‘strips the meaning from the experience’ (Bott Spillius, Milton, Garvey, Couve, & Steiner, 2011, p. 408). How reverie and containment can be offered to the therapist, or to the family, in the context of such anxieties, both real and fantasised, is an important question.

In a multidisciplinary CAMHS, one of the roles of the psychoanalytic psychotherapist is to offer opportunities for thought in a variety of spaces, such as multidisciplinary discussions, consultations, or while undertaking ‘urgent’ or ‘crisis’ assessments as part of a duty rota. In what follows, I have both roles in mind in my exploration of what psychoanalytic thinking can offer.

Salisbury and Baraitser (2020) frame psychoanalysis as an offer of a particular kind of time: ‘the offer of time and care, and of remembering, repeating and working through’ (p. 106, citing Freud, 1914). This is a time in which the psychoanalyst waits *with* the patient rather than *for* them, and thus ‘a treatment *of* or *with* time’: ‘Psychoanalytic care, in this account, functions through a form of prolonged waiting *with*’ (pp. 114, 115; original emphases). If psychoanalytic psychotherapy is an offer of time, this is also expressed through the rhythm of sessions: their dependable timing, duration and structure as they span the week (whether once-weekly or more frequent).

Yet if psychotherapy is regarded as inherently an offer of time (a ‘long-term’ intervention in the NHS context, even in its shorter manifestations), this brings another temporal consideration into play: urgency. How is anxiety to be managed enough to open up a space in which psychoanalytic psychotherapy – a treatment associated with the passing of time – can be offered? Can we afford to wait in the face of a teenage girl’s incessant self-harm, to accompany her on such a therapeutic journey? Can we afford not to wait, for the network around an impulsive teenage boy to settle sufficiently to enable him to take the first tentative steps towards recovery? (Note, here, how clinical metaphors for the therapeutic process are based on space and time.) With *risk* being part of the picture, an adolescent’s impatient response to an offer of psychotherapy - ‘I can’t wait that long’ (because I’ve got things to do) – although potentially healthy, easily turns into the parent’s or professional’s ‘we can’t afford to wait’ (for the psychotherapy to work, because the young person may die).

I am inclined to think that most of the risk management strategies mentioned above either represent a fantasy of a buying of time or a particular kind of management of the time of crisis. It is also common for clinicians to instruct parents to lock away anything dangerous in the house, such as sharp objects, medications and potential ligatures, and – this element usually implicit - to watch their child like a hawk. The indignity of this for the young person is often mitigated by framing it as a safety-net: a buying of time, while they are in a crisis, which can be dispensed with once they are in better mental health. As such, it may represent the playing out of conflicts between the young person and their parents about separation, togetherness and autonomy, although this is often not articulated.

To buy time is a curious concept, akin to the scholarship Salisbury and Baraitser (2020) and Baraitser and Brook (2020) cite about the importance of practices of care that use *waiting with* to create time. Paradoxically, they argue, such practices are particularly needed during crisis:

Crisis… may call less for judgement, and rather, paradoxically, call for the *suspension* of judgement. Here the offer would be of a form of care that *calls for no decision*, that appears to continue the state of crisis through the offer simply of more time. (Baraitser & Brook, 2020, p. 237; original emphasis)

This recognition that no decision and an offer of time may be more productive is echoed in the psychoanalytic literature on self-harm. Maria Papadima (2019), for instance, argues that clinicians need to pause for thought to consider the meaning of acts of self-harm, in the face of the clinical urgency such acts engender. Self-harm is thus intrinsically performative but also communicative; Anna Motz (2010) too underlines the communicative function of self-harm, to the extent that she argues that it should be seen as an expression of hope.

This brings us back to the question of whether, or under what conditions, a crisis may produce change. What is being suggested here is that such conditions may include the offer of time. It is precisely this suspension of action to create time that Bion offered in his reworking of Keats’s idea of ‘negative capability’ (Keats, 1817/1958, pp. 193-194):

Instead of trying to bring a brilliant, intelligent, knowledgeable light to bear on obscure problems, I suggest we bring to bear a diminution of the ‘light’- a penetrating beam of darkness; a reciprocal of the searchlight. (Bion, 1973-74 / 1990, p. 20)

As Laura Salisbury identifies, Bion’s ‘psychoanalytic commitment to slow process and practices of waiting’ (2020, p. 100) was partly informed by his experiences as a tank commander in World War I. She traces his early understanding of the ways in which populations can be held hostage by enemies who arouse infantile dread: ‘the field is held by *phantasies* of air-raids’ (Bion, 1940, p. 181). Bion recognised that solitary waiting can produce ‘nameless dread’ (1962, p. 116) and that communal effort may in extreme situations be called upon to manage such anxiety:

The [air-raid] alarm must be a call to action, and there must be an action to which every man and woman is called. (1940, p. 189)

This may shed some light on another function of *risk management* and *safety planning* for families and professionals: they call each person to action, in a way that feels collaborative and productive, and active rather than passive. Years later, the language of war permeates Bion’s imagery of thoughts and thinking (thoughts are ‘evacuated at high speed as missiles to annihilate space’; Bion, 1962, p. 113) as he works out a different way of managing thoughts:

For Bion, the only way to transform thoughts as an aerial bombardment to be managed through what he calls ‘evasion by evacuation’ [Bion, 1962, p. 117], is to suspend this mobilisation that seeks to rid the psyche of thoughts. Instead, analyst and patient must learn together how to wait and think … For Bion, such thinking, imagined according to the processes of a body able to absorb rather than be blown apart by bombs, indeed produces a space and time where violence can be held and thought about rather than enacted. (Salisbury, 2020)

Yet in seeking to offer time and thought amidst the violence - here, the violence of fantasised or actual suicidal acts rather than bombs - professionals, including those working psychoanalytically, must manage the nameless dread that I am calling the shadow of suicide.

Why the psychoanalytic offer of time is helpful in crisis situations, then, is surely that it allows a slowing of time, even in the midst of crisis. This is time in which the unconscious communication can be heard – in both the patient and the anxious professional. An example of this might be seen in the story of the boy who said he was ‘going to kill himself on Monday’, to which I reported the paradoxical reaction: ‘he’s going to kill himself, we need to get him in; oh fine, we’ve got till Monday’. Whatever the meaning of this communication for the young person in question (which is not my focus here), my own reaction might be understood as signifying a momentary but profound confusion about urgency, generated by the compelling claim on our time offered by this communication. The challenge here was perhaps to be sufficiently anxious but not overwhelmed by anxiety. Waiting until Monday itself would have been foolhardy, and might have communicated a dangerously literal interpretation of the boy’s words, or a reluctance to respond until absolutely necessary. Yet to insist that he be brought to the clinic immediately might have also been problematic. Not only would the clinic be near to closing for the evening, potentially diverting the young person and his parents to A&E, but such an offer might have been heard as indicating that we regarded his parents as unable to cope, his comment leading him straight to the specialists, bypassing them. Perhaps it would have also implied that there was no space for thought here, nor for ordinary loving care.

The need to strike such a balance, particularly in the face of intense anxiety, was noted by Melanie Klein in a paper written during World War II in which she explores the links between the individual’s internal world and the external horrors of war:

It becomes clear that … the incapacity to dissociate the evil father and parents from the good ones, to dissociate love from hate, and therefore to turn hatred against the evil thing – love and protection towards the loved and good people – that all this has a paralysing effect in the relation to external dangers…. If [the individual] feels to out-Hitler Hitler, then it will all end in complete destruction inside….I see fully confirmed … that death is terrifying to the utmost, if trust in internal relationships is weak. The danger may then be denied… or the individual becomes paralysed – which may amount to suicidal incapacity to deal with external dangers. (Klein, 1940, pp. 2-3)

In Salisbury’s précis:

If unprocessed internal phantasies could lead to psychic catastrophe and death through either a manic evasion of threat (not waiting long enough) or a dangerous paralysis (waiting too long). (Salisbury, 2020, p. 103)

The implication of offering time to young people who have self-harmed, or who have contemplated or attempted suicide, is that we attempt to wait with them in the face of this violence; that we do not ask them to eschew it. Yet as Campbell and Hale argue:

The explicit promise that the person will not try to kill themselves should be treated with extreme caution; suicidal impulses are devious and can trick both the person themselves and the professional wishing to help. To renounce the option of suicide is to lose a crucial sense of potency and self-efficacy. (2017, p. 87)

To hold the suicidal ideation in time and thought – a containment that is temporal, spatial and emotional – provides a space in which not only can these things start to be understood, but the passing of time can, hopefully, start to become less threatening. Exam pressure may turn out to be more manageable; adult life less daunting.

Perhaps ‘hopefully’ is the operative word here. For how can this offer of time be made in the face of the external pressures of urgency and risk and the internal ones of nameless dread (the shadow of suicide)? I would argue that what may be needed is to hold an authentically hopeful image in mind, alongside the violence. Supervision, as well as various kinds of reflective or supervisory spaces for the multidisciplinary team, are of course where the power of such contradictory thoughts and feelings can be held. Salisbury (2020, pp. 103-104) draws attention to the emphasis placed by Klein (though not associated with her in the popular imagination) on the role of genuinely good experiences in building up hope. Despite living during wartime, Klein wrote:

We look at nature, we read a book, we play with a child, we enjoy food, etc., and we have to remind ourselves that our life and country is at stake…. If the help provided by the fact that such good things we just enjoy exist, the belief in the good objects and in goodness ultimately, is not too much denial of the bad things, it may help us to take steps to preserve goodness externally, and may internally help us to remain calm in the face of danger. (1940, pp. 6-7)

To offer, then, a therapeutic waiting, when the young patient has so bleak an image of the future, surely requires the practitioner to hold onto an alternative image in the face of the nameless dread of suicide. This is the fantasy of a well patient: perhaps able to participate in ordinary teenage activities; perhaps a young adult who has shaken off the last vestiges of their adolescent torment. Such hopeful fantasies may be founded on clinical experience built up over the years. For this work to be meaningful, the safety plans need not to be ‘fragments [to shore] against my ruins’ but authentic expressions of commitment, care and time. Which alternative holds the most unconscious significance may be evidenced in the countertransference of the professional, which may allay fears that a safety plan is simply a ‘tick-box exercise’. The role of psychotherapy with such a young person, then, may be to allow rage, shame and destructiveness to be thought about, alongside glimmers of authentic hope.

16-year-old Jane, described above, continued to tell her psychotherapist how pointless life was, gradually starting to enumerate all the reasons why the future, albeit scarcely imaginable, was bleak. Over time, the psychotherapist realised that Jane’s psychiatrist, parents and social worker were reporting her better functioning at school and at home. Eventually Jane started to talk about an image she had formulated of herself going to university.

For young people like Jane, whose treatment holds them through the long wait to feel any better, the safety planning and risk-management ideally prove to be not just ‘fragments’ to shore against the ruins of impending catastrophe, but demonstrations of the family’s and the professionals’ commitment. This is a commitment to a network of relationships – between professional and family, family and child, professional and young person – in which the hope can truly be held. It is a commitment to *waiting for* and *waiting with*, their willing assumption of a protective function and their facing up to the bleakness and danger represented by the young person’s suicidal wishes. In the face of such catastrophic anxieties, that is no small thing.

## Footnotes

[1]
http://waitingtimes.exeter.ac.uk/

[2]
https://wellcomeopenresearch.org/collections/waitingtimes

[3] For thoughts on the way in which adolescent time may be seen as giving the lie to the halting of time in the Covid-19 lockdown, see Catty, 2020.

[4] All the vignettes used in this paper are composites: that is, they have been created from diverse clinical experiences drawn from years of practice. None of them represents a single real person.

[5]
https://zerosuicide.edc.org/

## References

[R1] American Psychiatric Association (2013). Diagnostic and statistical manual of mental disorders, Fifth Edition (DSM-5).

[R2] Baraitser L (2009). Maternal encounters: The ethics of interruption.

[R3] Baraitser L (2017). Enduring time.

[R4] Baraitser L, Brook W, Browne V, Danely J, Managhan T, Rosenow D (2020). Vulnerability and the politics of care. Proceedings of the British Academy.

[R5] Bion WR, Miller E (1940). The neuroses in war.

[R6] Bion WR (1962). The psycho-analytic study of thinking. International Journal of Psycho-Analysis.

[R7] Bion WR (1973-1974). Brazilian lectures.

[R8] Bott Spillius E, Milton J, Garvey P, Couve C, Steiner D (2011). The new dictionary of Kleinian thought.

[R9] Brannen J, Nilsen A (2002). Young people’s time perspectives: from youth to adulthood. Sociology.

[R10] Burton R, Faulkner TC, Kiessling NK, Blair RL (1621). The anatomy of melancholy.

[R11] Catty J (2020). Lockdown and adolescent mental health: reflections from a child and adolescent psychotherapist. Wellcome Open Research.

[R12] Campbell D, Hale R (2017). Working in the dark: Understanding the pre-suicide state of mind.

[R13] Cregeen S, Hughes C, Midgley N, Rhode M, Rustin M, Catty J (2017). Short-term psychoanalytic psychotherapy for adolescents with depression: A treatment manual.

[R14] Davies S (2019). Time and clinical governance in general practice.

[R15] Deleuze G, Boundas CV, Stivale C, Lester M (2004). The logic of sense.

[R16] Eliot TS (1922). Collected Poems 1909-1962.

[R17] Emanuel L (2006). A slow unfolding - at double speed: Reflections on ways of working with parents and their young children within the Tavistock clinic's under fives service. Journal of Child Psychotherapy.

[R18] Flexer MJ (2020). The ‘telegraphic schizophrenic manner’: Psychosis and a (non)sense of time. Time &Society.

[R19] Fonagy P, Rost F, Carlyle J, McPherson S, Thomas R, Fearon P, Goldberg D, Taylor D (2015). Pragmatic randomized controlled trial of long-term psychoanalytic psychotherapy for treatment-resistant depression: The Tavistock Adult Depression Study (TADS). World Psychiatry.

[R20] Freud S (1914). Remembering, repeating, and working through.

[R21] Freud S (1926). Inhibitions, symptoms and anxiety.

[R22] Goodyer I, Reynolds S, Barrett B, Byford S, Dubicka B, Hill J, Holland F, Kelvin R, Midgley N, Roberts C, Senior R (2016). Cognitive behavioural therapy and short-term psychoanalytical psychotherapy versus a brief psychosocial intervention in adolescents with unipolar major depressive disorder (IMPACT): a multicentre, pragmatic, observer-blind, randomised controlled superiority trial. Lancet Psychiatry.

[R23] Grupe DW, Nitschke JB (2013). Uncertainty and anticipation in anxiety: An integrated neurobiological and psychological perspective. Nature Reviews Neuroscience.

[R24] Karim F, Oyewande A, Abdalla LF, Ehsanullah RC, Khan S (2020). Social media use and its connection to mental health: A systematic review. Cureus.

[R25] Keats J, Rollins HE (1817/1958). The letters of John Keats.

[R26] Kelly Y, Zilanawala A, Booker C, Sacker A (2018). Social media use and adolescent mental health: Findings from the UK Millennium Cohort Study. EClinicalMedicine.

[R27] Klein M (1940). What does death represent to the individual?, 2: Melanie Klein Archive, Wellcome Library, PPKLE/C/96, 2018.

[R28] Midgley N, Ansaldo F, Target M (2014). The meaningful assessment of therapy outcomes: Incorporating a qualitative study into a randomized controlled trial evaluating the treatment of adolescent depression. Psychotherapy.

[R29] Midgley N, Parkinson S, Holmes J, Stapley E, Eatough V, Target M (2015). Beyond a diagnosis: The experience of depression among clinically-referred adolescents. Journal of Adolescence.

[R30] Minkowski E, Metzel N (1970). Lived time: Phenomenological and psychopathological studies.

[R31] Moore MD Appointment systems in post-war general practice: The affective life of temporal regulation in the British National Health Service, 1948-1980.

[R32] Motz A (2010). Self-harm as a sign of hope. Psychoanalytic Psychotherapy.

[R33] Neufeld SAS, Dunn VJ, Jones BP, Croudace TJ, Goodyer IM (2017). Reduction in adolescent depression after contact with mental health services: a longitudinal cohort study in the UK. Lancet Psychiatry.

[R34] Nuffield Trust (2019). Evidence for better health care. Hospital admissions as a result of self-harm in children and young people.

[R35] Papadima M (2019). Rethinking self-harm: A psychoanalytic consideration of hysteria and social contagion. Journal of Child Psychotherapy.

[R36] Royal College of Psychiatrists (2020). Technology use and the mental health of children and young people.

[R37] Salisbury L (2020). ‘Between-time stories’: Waiting, war and the temporalities of care. Medical Humanities.

[R38] Salisbury L, Baraitser L, Kirtsoglou E, Simpson B (2020). The time of anthropology: Studies of contemporary chronopolitics.

[R39] Sandford J, Loach K (1966). Cathy come home (Television drama).

[R40] Straus E, Broome MR, Harland R, Owen GS, Stringaris A (1928/2012). The Maudsley reader in phenomenological psychiatry.

[R41] Therrien A Fifth of 14-year-old girls in UK ‘have self-harmed’. BBC News website.

[R42] Turton P, McGauley G, Marin-Avellan L, Hughes P (2001). The Adult Attachment Interview: Rating and classification problems posed by non-normative samples. Attachment and Human Development.

[R43] Waddell M (2018). On adolescence: Inside stories.

[R44] Wajcman J (2015). Pressed for time: The acceleration of life in digital capital.

[R45] Wong PS (1999). Anxiety, signal anxiety, and unconscious anticipation: Neuroscientific evidence for an unconscious signal function in humans. Journal of the American Psychoanalytic Association.

